# A Few Plain Directions for Conducting the Chemical Analysis of Vegetable Bodies, Particularly Bark, Roots, &c. with a View to Their Introduction into the Practice of Medicine

**Published:** 1822-06

**Authors:** 


### MEDEOGRAPHY.

**Art. VII.**—*A few plain Directions for conducting the Chemical Analysis of Vegetable Bodies, particularly Bark, Roots, &c. with a View to their Introduction into the Practice of Medicine.* By the Editor.

As a supplementary article to the valuable publications of my friends, Dr. Paris and Mr. A. T. Thomson, on practical Pharmacology, it has long ago struck me that some instructions might be given to the readers of both those excellent works, for examining and analyzing any new vegetable substance, particularly bark of trees, roots, &c. that might be offered for practical purposes to the profession.

I could have wished that the task of drawing-up such instructions had fallen into better hands; but, as I have looked in vain in all the English publications, either purely chemical or chemico-pharmaceutical, for the accomplishment of this *desideratum*, I have deemed it no presumptuous undertaking in me to supply the deficiency, by enumerating the different processes I have been taught to follow in the analysis of vegetable bodies by my much-respected friends and preceptors, M. Vauquelin and the late Cadet de Gassicourt.

The first steps to be taken consist in examining the new vegetable body *physically*; and next as to its *chemical* properties.

The colour, taste, flavour, smell, external appearance, botanical and physiological characters, state of aggregation, density, &c. belong to the former inquiry: while the experiments made, with various tests, on the infusion, decoction, extract, and tincture, of the bark, root, &c. under examination, belong to the latter.

In describing the substance, and giving an account of the chemical experiments made with it, the same method should be adopted which has been followed during its analysis; and that method may be thus enunciated in the shape of general and aphoristic formulæ.

### A. Physical Characters.

1. Colour of the bark, root, &c.
2. Nature of its epidermis.
3. Thickness of the bark and the epidermis, individually.
4. Mode in which they adhere together.
5. Whether any of the woody fibres adhere to the epidermis.

6. Smell. Flavour. Perfume. Taste, permanent or evanescent.
7. Has the epidermis the same characters with the *liber?*
8. Is the bark easily powdered?
9. Does it readily attract moisture when exposed to the air?
10. Specific gravity of the bark.
11. Specific gravity when powdered.

### B. Chemical Characters.

#### *a. Infusion.*

1. A given quantity of the bark, coarsely powdered, infused in a sufficient quantity of cold water for a given number of hours.

2. Filter. Weigh the filtered infusion and the dry residuum on the filtre. Examine the smell, taste, colour, and flavour, of the infusion.

3. The filtered infusion should now be tried by

- *a.* Isinglass.
- *b.* Tartarized antimony.
- *c.* Prussiate of potash.
- *d.* Nitrate of barytes.
- *e.* Oxalic acid.
- *f.* Sulphate of iron.
- *g.* Supersulphate of alumine.
- *h.* Sulphate of copper.
- *i.* Oxalate of ammonia.
- *k.* Gallic acid.
- *l.* Lime-water.
- *m.* Tincture of galls.
- *n.* Turmeric paper.
- *o.* Litmus paper.

4. Mark, in writing, the result of each experiment, and of every precipitate that follows; dry the latter, weigh them, and keep them for further examination.

#### *b. Decoction.*

1. A given weight of the bark, or root, coarsely powdered, boiled in a sufficient quantity of distilled water, to the reduction of one-third.

2. Filter the decoction. Mark its colour, taste, flavour, perfume.

3. Test it with the same re-agents, and note the results; and whether or not they be similar to those obtained in treating the infusion.

#### *c. Extract.*

1. The same given quantity of bark, root, or plant, coarsely powdered, is to be subjected to the action of boiling water several successive times. The different decoctions added together are to be slowly evaporated, in large vessels, to the consistence of extract. The extract to be weighed; the colour, taste, &c. noted.

3. Ascertain how much of the extract is dissolved in alcohol. Mark the taste, colour, and smell, of the resulting tincture.

4. Test the tincture by

- *a.* Distilled water.
- *b.* Emetine, gelatine, litmus and turmeric paper, oxalic acid, carbonate of potash; and write down the result.
- *c.* Sulphate of iron.
- *d.* Nitrate of barytes.
- *e.* Nitrate of mercury.
- *f.* Nitrate of silver.
- *g.* Prussiate of potash.

5. If any residuum be left after treating the extract with alcohol, subject it to the action of distilled water, exposing it to a gentle heat at the same time, and trying the solution, if any, with the above tests.

### *d. Alcoholic Infusion.*

1. A given weight of the bark, root, &c. coarsely powdered, is to be infused into a known quantity of alcohol for a given number of days.

2. The smell, colour, taste, perfume, &c. of the resulting infusion are to be noted.

3. The infusion is to be tested with

- *a.* Distilled water.
- *b.* Gallic acid.
- *c.* Sulphate of iron.

4. The residuum, after filtering the alcoholic infusion, is to be weighed, and examined.

### *e. Distilled Tincture.*

1. The alcoholic infusion should be next distilled in a glass retort.

2. Observe whatever phenomena may occur during the distillation.

3. The result of the distillation should be tested with

- *a.* Distilled water.
- *b.* Prussiate of potash.
- *c.* Sulphate of iron.

4. Dry the residuum; weigh it, note its colour and consistency, and whether it attracts moisture.

5. Is it soluble in water?

6. Is it soluble in alcohol, ether, oil of turpentine, caustic alkaline solution?

7. In either case, try the solution with the usual re-agents.

